# Building evidence-based nursing competence through Class-Clinic-OBE in postgraduate education: a quasi-experimental study

**DOI:** 10.1186/s12912-026-04981-6

**Published:** 2026-07-28

**Authors:** Jinqiong He, Jin Li, Weilin Lin, Di Tian, Yuxia Lin, Hui Li, Qiao Wang, Xi Su

**Affiliations:** 1 Guangzhou Medical University, No. 1 Xinzao Road, Xinzao Town, Panyu District, Guangzhou, Guangdong 511436 China; 2 Southern Medical University Hospital of Integrated Traditional Chinese and Western Medicine, Southern Medical University, No. 13 Shi Liu Gang Road, Haizhu District, Guangzhou, Guangdong 510315 China

**Keywords:** Postgraduate education, Evidence-based nursing, Situated learning theory, OBE, Learning mode

## Abstract

**Background:**

Postgraduate nursing education requires Evidence-based Nursing (EBN) competence to train professionals who can synthesize evidence and translate it into clinical practice. However, current EBN education often relies on one-way knowledge transmission in decontextualized classrooms. This approach fails to bridge the gap between theory and practice. To address this fundamental issue, guided by Situated Learning Theory (SLT) and Outcome-Based Education (OBE), this study designed the Class-Clinic-OBE learning mode and evaluated its effectiveness in improving nursing postgraduates’ EBN competence and academic performance.

**Methods:**

A quasi-experimental, non-concurrent two-cohort study was conducted. It included 151 nursing postgraduates during the autumn semester of 2023 and 2024. The comparison group (2023 grade: *n* = 72) followed Mixed Learning, while the intervention group implemented the Class-Clinic-OBE mode (2024 grade: *n* = 79), which integrated theoretical and experimental learning in “The First Classroom” with clinical practice through “Field-Based Activities.” Outcomes were assessed through two primary measures. EBN competence was measured using the Evidence-Based Practice Competence Questionnaire (EBP-COQ) across three assessment time points (T0: Pre-class, T1: After-The First Classroom, and T2: After-Field-Based Activities). Academic performance was evaluated through offline and online channels. Offline assessments included scores from proposal presentations, final presentations, and preliminary draft submissions. Online performance was tracked using engagement data from the Chaoxing platform. The data were analyzed using descriptive and inferential statistical techniques in SPSS 27.

**Results:**

EBN competence in the Class-Clinic-OBE group showed a significant time effect across the three assessment points [*F*(2,150) = 4.553, *p* = 0.012]. Post hoc tests revealed that EBN competence at T2 (107.50 ± 12.01) was significantly higher than at T0 (103.99 ± 12.30, *p* = 0.023) and T1 (104.14 ± 10.35, *p* = 0.037). A significant group × time interaction was observed for academic performance, with the Class-Clinic-OBE group showing greater improvement from proposal to final presentation than the Mixed Learning group [*F*(1,149) = 13.418, *p* < 0.001, η² = 0.083].

**Conclusions:**

The Class-Clinic-OBE learning mode supports the progressive development of EBN competence through structured cross-context learning and outcome-oriented assessment. This research provides educators with a feasible framework for integrating EBN theory into postgraduate curriculum design.

**Trial registration:**

Not applicable.

**Supplementary Information:**

The online version contains supplementary material available at https://doi.org/10.1186/s12912-026-04981-6.

## Introduction

### Background

Evidence-based Nursing (EBN) competence is the comprehensive ability that integrates the best available research evidence, clinical expertise, and patient preferences to support nursing practice [1]. Developing EBN competence has become an important focus in postgraduate nursing education [2]. In complex healthcare environments, nursing practitioners are required to do more than follow routine care protocols. They are increasingly expected to retrieve, critically appraise, and translate research evidence into clinical practice [3]. This expectation is particularly relevant for nursing postgraduates. They are regarded as future advanced nursing practitioners. Their professional responsibilities include leading evidence-based practice, promoting nursing innovation, and facilitating healthcare improvement in complex clinical settings [4]. Therefore, it is essential for nursing postgraduates to develop this competence. It enables them to support clinical decision-making and deliver high-quality nursing care.

However, developing this essential competence among nursing postgraduates is challenged by two main educational limitations. First, there is a disconnect between classroom learning and clinical practice in EBN education. Previous studies have shown that opportunities for sustained participation in authentic clinical situations remain limited in EBN education [5]. However, becoming a competent evidence-based practitioner requires more than theoretical knowledge [6]. It also requires students to engage with authentic clinical problems. Through this process, they learn how evidence can be translated into clinical decision-making and practice improvement. Existing EBN education approaches mainly rely on classroom-based learning [7]. After coursework, students are rarely provided with sustained opportunities to apply evidence in clinical practice. As a result, their learning often remains decontextualized. This may make it difficult for them to translate theoretical knowledge into competent performance when confronted with the complexity of actual patient care. Although simulation and case-based learning may partially address this issue [8], they cannot fully substitute for authentic clinical participation. Second, current educational assessment approaches may not fully align with the developmental nature of EBN competence. This competence develops progressively through continuous learning, practice, reflection, and feedback [9]. However, existing studies suggest that assessment of EBN competence is still commonly based on isolated, summative metrics, such as final exam scores [10, 11]. Such approaches provide limited insight into students’ competence development over time. Consequently, educators may find it difficult to fully understand students’ developmental trajectories. At the same time, opportunities for students to receive continuous feedback throughout the learning process may remain limited.

To address these limitations, this study designed a cross-context learning mode (Class-Clinic-OBE). The intervention extends EBN learning from the classroom to clinical situations while enabling dynamic tracking of competence development over time. Therefore, this study aimed to evaluate its effects on improving EBN competence and academic performance among nursing postgraduates.

### Research question

1. What is the impact of the Class-Clinic-OBE learning mode on nursing postgraduates’ EBN competence?
2. What is the impact of the Class-Clinic-OBE learning mode on nursing postgraduates’ academic performance?

### Theoretical framework

From the perspectives of Situated Learning Theory (SLT) and Outcome-Based Education (OBE), the Class-Clinic-OBE learning mode was designed.

Situated Learning Theory is a core educational theory proposed by Jean Lave and Etienne Wenger in 1991 [12]. It suggests that learning is inherently social and develops through active participation and social interaction within authentic situations [13]. The core principles of SLT include Situated Learning, Legitimate Peripheral Participation (LPP), Communities of Practice (CoPs), and Identity Transformation [14]. (1) Situated Learning: Knowledge acquires meaning through the activities, culture, and situation in which it is developed and used. (2) LPP: Learning starts with simple, low-risk tasks at the periphery of a community. Learners gradually advance to full participation as their expertise develops. (3) CoPs: Learning occurs through active participation with a group sharing a common profession or interest, where regular interaction fosters sharing and learning. (4) Identity Transformation: Through gradual participation, identities evolve from learners to competent practitioners who master practical application. SLT is now commonly used to design learning experiences that support the development of clinical competence through engagement in authentic practice contexts [15–17]. Growing evidence suggests that SLT-guided educational approaches enhance students’ integration of theory and practice [18–20].

Outcome-Based Education is a learner-centered educational approach introduced by William Spady in 1994 [21]. It emphasizes that learning outcomes drive the entire curriculum design. Students are expected to achieve clear, measurable outcomes upon completion. The core principles of OBE are Student-Centered, Outcome-Based, and Continuous Improvement [22, 23]. (1) Student-Centered: Curriculum design and implementation focus on what students actually achieve. (2) Outcome-Based: Curriculum design begins with the specific learning outcomes students must demonstrate. Instruction and assessment are then designed backward from these outcomes. (3) Continuous Improvement: Feedback and assessment support continuous refinement of curriculum design and implementation. This process enhances educational quality and facilitates the achievement of desired outcomes. OBE is an important approach in nursing education reform [24]. Previous studies have demonstrated that OBE-guided educational approaches can support competence-based curriculum design, outcome-aligned assessment, and continuous quality improvement [25–27].

In the Class-Clinic-OBE learning mode, OBE provides the teleological “what for,” and SLT provides the procedural “how” of learning. Within the OBE framework, enhancing EBN competence is established as the overarching educational objective. This objective provides clear direction for backward curriculum design. Students engage in learning activities structured to develop competence in searching, critically appraising, and translating evidence. Finally, a preliminary draft derived from the application of practice-based evidence is designated as the key learning outcome. This draft is a tangible product of students’ ability to translate evidence into clinical practice.

SLT achieves this outcome through collaborative EBN projects within CoPs. Students first engage in classroom-based CoPs with supervisors and senior postgraduates, beginning as peripheral learners through structured tasks (e.g., literature searches, quality assessment of literature). They then progress to clinical CoPs (including clinical tutors and advanced practitioners) to adapt and implement evidence in response to real patient needs. As students move from classrooms to clinical communities, they iteratively develop and refine a practice-based preliminary draft. This progression mirrors LPP and catalyzes an identity transformation from peripheral learners to emerging evidence-based practitioners. Simultaneously, OBE’s continuous improvement principle is embedded through iterative feedback and longitudinal assessment. Students receive assessment and feedback at three key milestones, including proposal presentations, final presentations, and preliminary drafts. Longitudinal assessment enables ongoing monitoring of students’ progress in evidence searching, critical appraisal, evidence application, and academic writing. Iterative feedback helps students identify deficiencies and refine their EBN projects. Through repeated cycles of assessment, feedback, and revision, students progressively develop and consolidate their EBN competence. Thus, both theories work synergistically to address the two major limitations identified in current EBN education. OBE defines what students must achieve and supports longitudinal assessment and continuous improvement, whereas SLT facilitates the transition of learning from the classroom to clinical practice.

### Learning mode

**Fig. 1:**
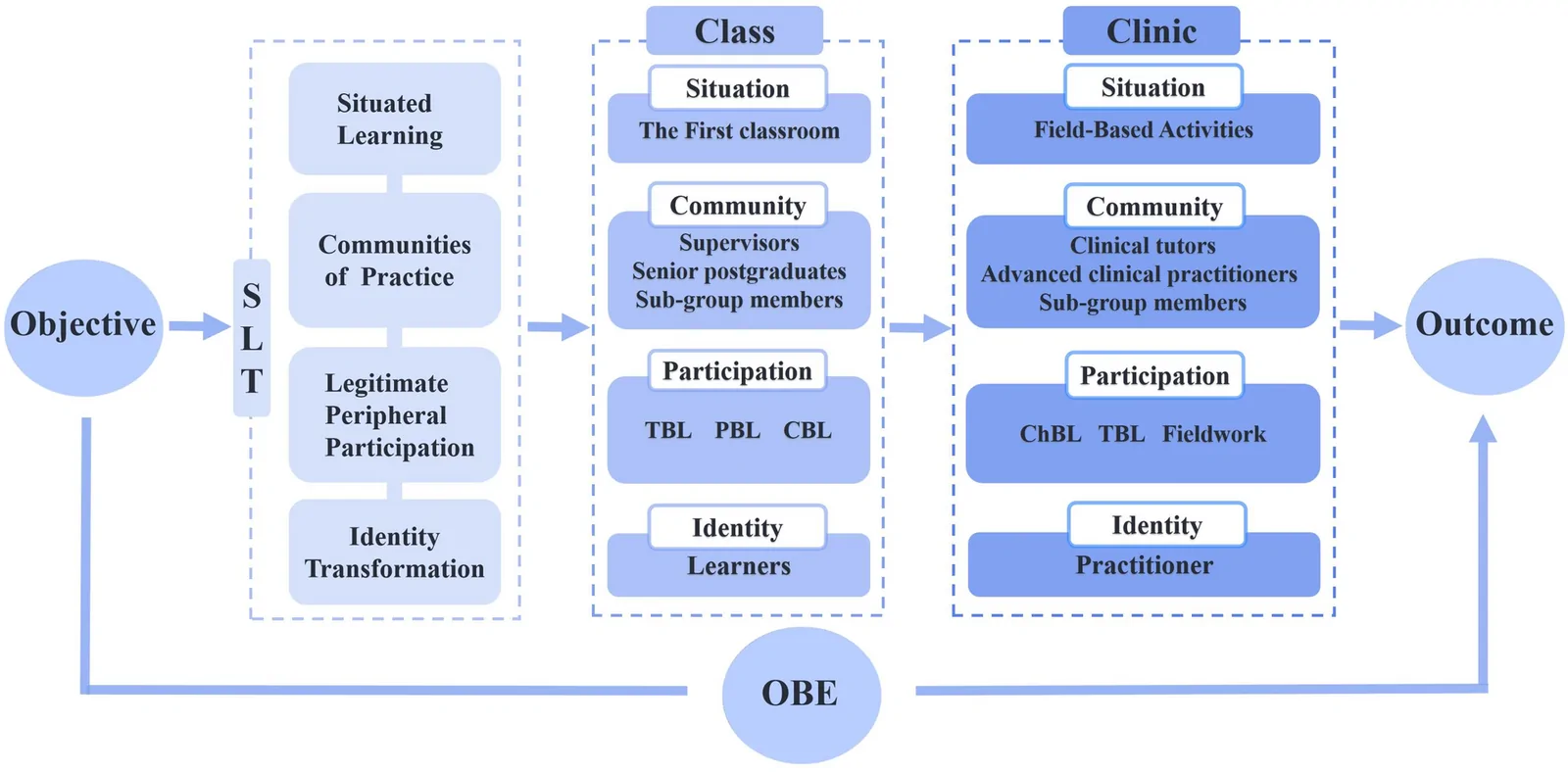
Design of the Class-Clinic-OBE learning mode

The Class-Clinic-OBE learning mode integrates SLT and OBE into a structured, phased instructional process that spans classroom and clinical settings. It is designed to address two major limitations in EBN education: situational disconnection and assessment inadequacy. As illustrated in Fig. 1, the mode supports the initial transition from classroom to clinical practice through cross-context learning. Each situation is characterized by clearly defined participants, learning activities, and roles, and is guided by the backward design of OBE and continuous assessment.

The Classroom Situation, also referred to as “The First Classroom,” represents the foundational learning stage that aligns with the nine advanced themes of the EBN curriculum. At this stage, students are organized into fixed subgroups and engage in collaborative EBN project learning. Learning activities are conducted within simulated clinical situations using Team-Based Learning (TBL), Project-Based Learning (PBL), and Case-Based Learning (CBL). Supported by the Chaoxing platform for online resource sharing and timely offline guidance from CoPs, this stage aims to develop students’ foundational evidence-based knowledge and collaborative competence.

Following completion of one semester of coursework, students progress to the Clinic Situation (“Field-Based Activities”) in accordance with the postgraduate training program. In this stage, they gain practical experience in authentic clinical contexts. Guided by both clinical tutors and advanced clinical practitioners, postgraduates initially adapt their EBN projects through Challenge-Based Learning (ChBL) and fieldwork in the practicum department. Initially occupying peripheral roles, students gradually assume greater responsibility for implementing evidence-based projects in clinical settings, thereby facilitating a transformation from learners to practitioners. This stage focuses on evaluating the clinical application of EBN projects and the development of professional identity.

## Methods

### Aim and design

A non-concurrent two-cohort quasi-experimental design was used in this study to evaluate the effectiveness of the Class-Clinic-OBE learning mode in the EBN course for postgraduates. The study was not registered because it did not meet the criteria for a randomized controlled trial.

### Setting and participants

This study was conducted at the School of Nursing of a medical university in Guangzhou, China. The research involved two groups of full-time master’s nursing postgraduates in the academic years of 2023 and 2024.

A total of 151 nursing postgraduates enrolled in the EBN course participated in the study. The course was delivered during the first academic term and aimed to develop students’ EBN competence for clinical nursing practice. The teaching team consisted of experienced educators and practitioners in EBN, including full-time academic supervisors and clinical tutors. To ensure consistency in instruction, the same teaching team taught both groups. The comparison group, consisting of 72 students enrolled in the 2023 academic year, received instruction via a Mixed Learning Mode. The intervention group, consisting of 79 students enrolled in the 2024 academic year, was taught using the Class-Clinic-OBE learning mode. The effectiveness of this novel mode is determined by evaluating postgraduates’ EBN competence and academic performance at multiple time points throughout the course.

### Intervention

The EBN course was designed to encompass 36 class hours, each lasting 40 min, and delivered over a 9-week curriculum. The course allocated 16 class hours to theoretical classes and 20 h to experimental classes. Both the Mixed Learning group and the Class-Clinic-OBE group completed the course as planned.

### Mixed learning (the comparison group)

The Mixed Learning Mode included five main components: (1) objectives, (2) content, (3) methods, (4) evaluation, and (5) resources [28]. (1) Objectives: To equip students with the essential knowledge, skills, and literacy required for EBN. (2) Content: The course covered five fundamental themes. The first theme: Overview of EBN, Defining Evidence-Based Questions and Searching for Evidence. The second theme: Appraising and Grading of Evidence and Assessing the Quality of Literature. The third theme: Learning Systematic Review and Meta-analysis Using RevMan Software. The fourth theme: Learning and Presentations of Qualitative Research Meta-Synthesis. The fifth theme: Knowledge Translation, Evidence-Based Application Models, Clinical Practice Guideline. (3) Methods: A blended approach combining online and offline instruction was used. Online components included video lectures, reading materials, and chapter quizzes for self-paced learning. Offline sessions incorporated TBL, CBL, PBL, and ChBL methods to promote active learning and critical thinking for EBN application. (4) Evaluation: Both formative and summative evaluations were employed. Formative assessments involved evaluating students’ performance during the group project proposal, while summative evaluations were based on final project presentations. (5) Resources: A Small Private Online Course (SPOC) was developed on the Chaoxing platform (link: https://www.chaoxing.com/) for EBN. The SPOC included high-quality meta-analytical articles, software download links, instructional videos on data processing, and an array of supplementary materials.

### Class-Clinic-OBE (the intervention group)

**Fig. 2:**
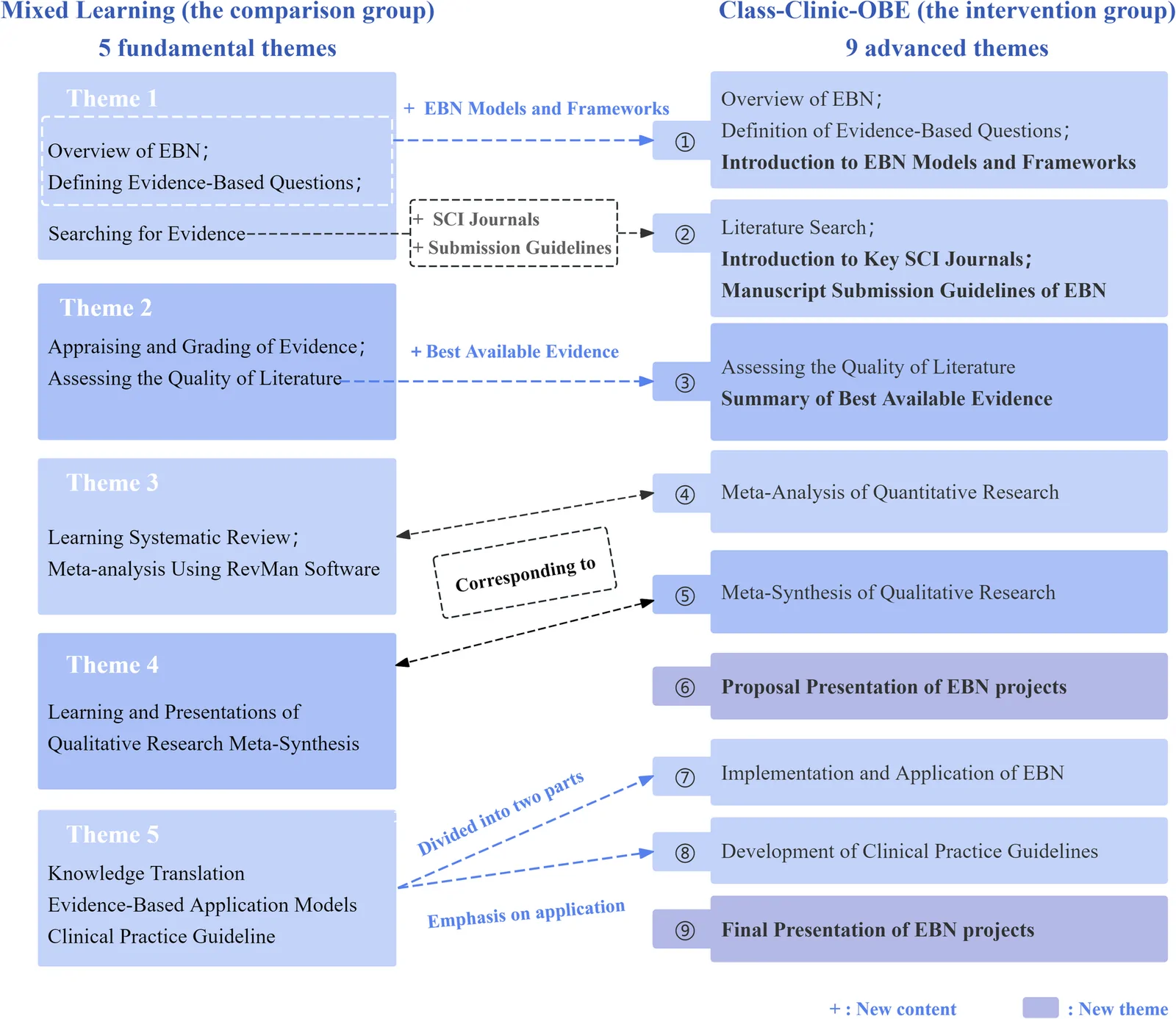
Comparison of thematic structures between the two groups

The intervention group employed a comprehensive learning approach, namely the Class-Clinic-OBE mode, which builds upon the Mixed Learning Mode implemented in the comparison group. This comprehensive approach emphasizes longitudinal development from classroom learning to clinical practice to help postgraduate students achieve true mastery in EBN theory and application. Specifically, the Class-Clinic-OBE group received expanded thematic content and Field-Based Activities within authentic clinical situations. Compared to the five fundamental themes in the Mixed Learning group, the Class-Clinic-OBE group adopted a more in-depth thematic structure, subdivided into nine advanced themes, as shown in Fig. 2. Furthermore, to accommodate the broader theme scope, the Class-Clinic-OBE group also implemented a teaching organization that integrated “The First Classroom” and “Field-Based Activities”. The term “The First Classroom” refers to conventional classroom instruction, primarily including textbook-based theoretical classes and group-conducted experimental sessions. “Field-Based Activities” involves an off-campus clinical practicum. Besides, unlike the mixed evaluation method employed in the Mixed Learning group, the Class-Clinic-OBE group adopted a multidimensional, dynamic evaluation system. This system included EBN competence scale scores measured at three distinct time points, offline academic performance metrics derived from the EBN projects (proposal presentations, final presentations, and preliminary drafts), and online performance data collected on the Chaoxing platform. As delineated in Table 1, the details of the teaching organization form of the EBN course are presented (Table 1). This integrated instructional design initially bridges classroom learning and clinical application. Table 2 provides an overview of the key learning methods used at each stage of the Class-Clinic-OBE mode (Table 2).

**Table 1:** Teaching organization form of the EBN course

Advanced Theme	Progressive Situation	Project-based Teaching Organization Form	Multidimensional Evaluation
① Overview of EBN; Definition of Evidence-Based Questions; Introduction to EBN Models and Frameworks. ② Literature Search; Introduction to Key SCI Journals and Manuscript Submission Guidelines of EBN. ③ Assessing the Quality of Literature; Summary of Best Available Evidence. ④ Meta-Analysis of Quantitative Research. ⑤ Meta-Synthesis of Qualitative Research. ⑥ Proposal Presentation of EBN projects. ⑦ Implementation and Application of EBN. ⑧ Development of Clinical Practice Guidelines. ⑨ Final Presentation of EBN projects.	**Class**: **The** **First** **Classroom** = Theoretical classes 16 class hours 4 themes ① ⑤ ⑦ ⑧ ་ Experimental classes 20 class hours 5 themes ② ③ ④ ⑥ ⑨	**Pre-Class (Chaoxing online platform)** **•Course Guidelines**: Released before the EBN course begins to help students become familiar with the schedule and how they will be evaluated. **•Courseware (Task Points)**: Uploaded before each class so that students can preview the content. **In-Class** **•Class Attendance**: Students need to scan the QR code on-site to check in on the Chaoxing platform. **•Teachers give lectures** in theoretical classes. **•Constructing communities of practice (CoPs) to accomplish EBN projects** in experimental classes. Details are below: •**Selecting the type of EBN projects freely**: Based on their own interests or their supervisors’ research direction, students can freely select one of the following types: Summary of Best Available Evidence, Meta-Analysis of Quantitative Research or Meta-Synthesis of Qualitative Research. •**Establishing the subgroups of EBN projects**: Students are divided into 3 groups based on their selection from the three types above. Each group is then subdivided into 12 smaller subgroups to collaborate. Each subgroup includes 2–3 members. •**Forming CoPs of EBN projects: 1 supervisor + 2 senior postgraduates + 2–3 subgroup members.** **Supervisors** provide overall guidance on EBN knowledge, resources, and skills throughout the project accomplishment process. **Senior postgraduates** attend practice sessions to provide timely assistance with operational tasks. **Subgroup members** collaborate to accomplish project presentations. **After-Class (Chaoxing online platform)** **•Module Quizzes**: Adopting a level-based, gamified quiz for gradual, in-depth learning. **•Extended Resources**: Providing knowledge graphs, software download links, high-quality meta-type articles, and teaching videos about literature management and data processing.	**•EBN competence (measured by the EBP-COQ scale)** •**Pre-Class**: Measurement taken for the first time. •**After-The first classroom**: Measurement taken for the second time. **•Academic performance** **(offline)** •**proposal presentation and final presentation**: Supervisors use self-made grading scales to assess CoPs’ project accomplishments. The concrete grading criteria and requirements are uploaded on the Chaoxing platform in advance. **•Academic performance** **(online)** •**engagement on the Chaoxing platform**: Task Points (50%) + Module Quizzes (40%) + Class Attendance (10%).
Clinical Practicum (In accordance with the nursing postgraduates’ training programme, students progress to the clinical practicum upon completion of classroom coursework)	**Clinic**: **Field-Based Activities** (occurred within the first month of clinical practicum)	**In-Clinic** •**Constructing CoPs to adapt EBN projects to clinic situation: 1 clinical tutor + 2 advanced Clinical Practitioners + 1 postgraduate.** **Clinical tutors** coordinate the department’s execution and offer timely guidance. **Advanced clinical practitioners** organize meetings and give patients information. **Postgraduates** take charge of communication and practice projects. •**Adaptation**: The CoPs attempt to preliminarily adapt the results of EBN projects to specific clinical nursing processes and refine the projects’ outcomes based on actual clinical needs. •**Submission**: Ultimately, the EBN project plan of each subgroup needs to be converted into an evidence-based preliminary draft integrating theory and clinical practice.	•**EBN competence (measured by the EBP-COQ scale)** **•After-Field-Based Activities**: Measurement taken for the third time. •**Academic performance** **(offline)** **•Preliminary draft**: Supervisors use another self-made grading scale to assess draft quality. The concrete grading criteria and requirements are uploaded on the Chaoxing platform in advance.

Each EBN project group is subdivided into 12 subgroups, for a total of 36 EBN practice subgroups

Each EBN project group is assigned one faculty supervisor, who serves as the sole rater for all 12 subgroups and independently evaluates their academic performance across proposal presentations, final presentations, and preliminary drafts

Abbreviations: EBN, Evidence-Based Nursing; OBE; SCI, Science Citation Index; CoPs, Communities of Practice; EBP-COQ, Evidence-Based Practice Competency Questionnaire

**Table 2:** Detailed learning strategies of the Class-Clinic-OBE mode

Learning Content	Learning Stage	Learning Strategy	Corresponding Outcome
The First Classroom ①⑤⑦⑧	Pre-Class	Review the course guidelines and teaching schedule. Preview courseware for each theme.	Task Points
	In-Class	**Teachers deliver lectures**: 120 min. **CBL**: 40 min. Use published meta-analysis articles to inform the discussion.	Class Attendance
	After-Class	Five fundamental test questions for each theme. Resources: Knowledge graphs and articles (various classic systematic meta-analysis papers; clinical practice guidelines).	Module Quizzes
The First Classroom ②③④	Pre-Class	Preview courseware for each theme. Install the EndNote and RevMan analysis software in advance using the provided link resource.	Task Points
	In-Class	Conducting communities of practice (CoPs). **CBL**: 80 min. Use published meta-analysis articles’ data and self-made teaching videos to study literature search, quality assessments, meta-analysis, and the detailed operations of EndNote and RevMan. **TBL + PBL**: 80 min. Apply learned methodologies for data analysis and chart creation to each CoP’s own EBN projects, under the on-site guidance of supervisors and senior postgraduates.	Class Attendance
	After-Class	The CoPs must hold a weekly group meeting for discussion. Five fundamental test questions for each theme. Resources - Articles: Quality assessment tools and usage methods; Chinese and English database search guidance and tutorials on Boolean logic operators. Resources - Videos: Database literature search and EndNote literature import instructional videos; RevMan operation videos.	Module Quizzes
The First Classroom ⑥⑨	Pre-Class	Review the self-made grading scale and grading criteria.	Task Points
	In-Class	**TBL + PBL**: 160 min. Students present the progress and completion of their self-selected EBN projects and receive grading and feedback from supervisors.	Class Attendance
	After-Class	Refine and modify the evidence-based project based on the supervisor’s feedback to lay the groundwork for subsequent implementation in clinical practice.	Proposal Presentation and Final Presentation
Field-Based Activities (Students’ assigned hospital affiliation primarily determines the hospital where a student conducts their practicum and EBN project)	Preparation (Identify the gap between evidence and practice)	**TBL**: 1 week. The CoPs must hold a weekly group meeting for discussion and feedback. Compare current departmental protocols with evidence-based practice projects to identify gaps, and collaboratively develop a detailed, step-by-step implementation plan.	Preliminary Draft
	Adaptation (Assess barriers, and develop actionable protocols and standards)	**ChBL + Fieldwork**: 3 weeks. Under the close guidance of clinical tutors, pilot the new evidence-based protocol within a small-scale setting. Collect process and outcome data post-pilot and compare with baseline to assess preliminary effectiveness. Each participating hospital will select 1–2 key projects that demonstrate successful implementation and strong innovation for additional resources to support in-depth development and publication of outcomes. Key projects such as: Effects of perineal massage at different stages on perineal and postpartum pelvic floor function in primiparous women: a systematic review and meta-analysis. Summary of the best evidence on hemostasis outcomes following neonatal blood collection using different pressure methods.	

①-⑨ refers to 9 advanced themes, as detailed in Table 1

Each theme covers 4 class hours, each lasting 40 min (a total of 36 class hours)

Abbreviations: EBN, Evidence-Based Nursing; OBE, Outcome-Based Education; CoPs, Communities of Practice; CBL, Case-Based Learning; TBL, Team-Based Learning; PBL, Problem-Based Learning; ChBL, Challenge-Based Learning; SPOC, Small Private Online Course; PICO, Patient, Intervention, Comparison, Outcome; RevMan, Review Manager; SCI, Science Citation Index

### Primary outcome

The primary outcome was EBN competence, measured by the Evidence-Based Practice Competency Questionnaire (EBP-COQ). Originally developed by Ruzafa-Martinez [29], the questionnaire was linguistically adapted for Chinese populations by Li Min [30] through rigorous cross-cultural validation procedures. The Chinese version demonstrated robust psychometric properties, with a content validity index (CVI) of 0.84 and excellent internal consistency (Cronbach’s α = 0.931). The 25-item questionnaire includes three distinct dimensions: attitude, skills, and knowledge. Each item uses a 5-point Likert scale from 1 (strongly disagree) to 5 (strongly agree), yielding a total possible score of 25–125. Higher scores indicate better EBN competence. The original English version of the full EBP-COQ is provided in Appendix Material 1.

### Secondary outcome

The secondary outcomes included offline academic performance in the EBN projects (proposal presentations, final presentations, and preliminary drafts) and online academic performance on the Chaoxing platform. The multidimensional learning evaluation system consisted of the four components mentioned above. Online performance was tracked using engagement data from the ChaoXing platform. In the offline evaluation, each academic performance component was assessed using structured self-made grading scales with distinct domains and weighting criteria tailored to each assessment. Proposal presentations primarily assessed the innovation and feasibility of the evidence-based projects (35%). Final presentations evaluated the quality of implementation and academic rigour (35%). Preliminary drafts served as the final output of the EBN projects and the foundation for future publications (20%). Notably, the preliminary draft was treated as an exploratory outcome within the OBE framework and was therefore assessed only in the Class-Clinic-OBE group. Engagement grades on the Chaoxing platform reflected students’ independent learning (10%).

### Data collection

EBN competence: The survey included sociodemographic information and the EBP-COQ scale. Identical questionnaires were distributed to all participants in both the Class-Clinic-OBE and Mixed Learning groups via the Questionnaire Star online platform (https://www.wjx.cn/). To monitor developmental trajectories, the Class-Clinic-OBE group was evaluated across three assessment time points during the 13-week study. T0 (Pre-class): Evaluated before any EBN course instruction began. T1 (After-The First Classroom): Evaluated immediately following the completion of all classroom-based instruction in The First Classroom, which occurred at the end of the 9-week course. The interval between T0 and T1 was 9 weeks. T2 (After-Field-Based Activities): Evaluated after completing the first month of Field-Based Activities, approximately 4 weeks after T1. Because equivalent longitudinal measurements were not collected at all corresponding assessment time points in the comparison group, EBN competence tracking was performed only in the Class-Clinic-OBE group.

Offline academic performance: To ensure comparability, the same self-made grading rubrics were used for both groups to assess academic performance (proposal presentation and final presentation). Detailed scoring criteria are provided in Appendix Material 2. In addition, the Class-Clinic-OBE group produced an additional academic outcome (preliminary drafts) following the Field-Based Activities. A specific rubric was accordingly developed for this outcome, see Appendix Material 3. All rubrics were aligned with curriculum objectives and tailored to different types of EBN projects (Summary of Best Available Evidence, Meta-Analysis of Quantitative Research or Meta-Synthesis of Qualitative Research). At first, all supervisors underwent a trial scoring exercise using identical, anonymized samples of previous student work to familiarise themselves with the grading rubrics. The scoring results were then subjected to a thorough, methodical discussion, with each result examined in turn. This process was continued until a high degree of consensus was achieved on the understanding and application of all the criteria. In the formal assessment, each EBN project group was evaluated by a single designated supervisor, who served as the sole rater for all 12 subgroups. All grading rubrics and requirements are uploaded in advance on the Chaoxing platform for students to review.

Online academic performance: Engagement data on the Chaoxing platform were exported directly from the online system. Final grades were calculated as 50% task points, 40% module quizzes, and 10% class attendance.

### Data analysis

All data were analyzed using the Statistical Package for Social Sciences (SPSS) software, version 27 (IBM Corp, Armonk, NY, USA). Categorical variables were presented as frequencies and percentages. The normality of continuous variables was assessed using the Shapiro-Wilk tests and Q-Q plots. Data that were normally distributed were expressed as the mean ± standard deviation (SD). Parametric or non-parametric tests were applied as appropriate based on data distribution. Independent-samples t-tests were used to compare age, chi-square tests were used to compare gender, and family residence. Repeated-measures analysis of variance (RM-ANOVA) was used to compare changes in academic performance over time between groups and to assess academic performance and EBN competence scale scores in the Class-Clinic-OBE group at different time points. The significance level was accepted at *P <* 0.05.

### Ethics approval and consent to participate

This study was approved by the Guangzhou Medical University Ethics Committee (reference number 202411018, dated September 1, 2024). All procedures were conducted in accordance with the principles of the Declaration of Helsinki. All participating teachers and nursing students provided written informed consent prior to the research. Participants retained the right to withdraw from the study at any time without penalty or consequence to their academic performance.

## Results

### Participant characteristics

There were 79 students in the Class-Clinic-OBE group, and 72 in the Mixed Learning group. After initial analysis, three students in the Class-Clinic-OBE group were excluded from the final analysis due to providing low-quality questionnaires, characterized by identical responses to all items. The final sample comprised 148 students, with 76 in the Class-Clinic-OBE group and 72 in the Mixed Learning group. No significant differences were found between the two groups at baseline in terms of gender, age, and family residence (all *P >* 0.05), indicating comparability (Table 3).

**Table 3:** Characteristics of the participants

Variables	Class-Clinic-OBE group (*n* = 76) *n* (%) or mean (SD)	Mixed Learning group (*n* = 72) *n* (%) or mean (SD)	χ² / t	*p*
Gender				
Male	6 (7.9%)	6 (8.3%)	0.010	0.922
Female	70 (92.1%)	66 (91.7%)		
Age	23.11 (2.06)	23.69 (1.77)	1.866	0.064
Family residence				
City	39 (51.3%)	37 (51.4%)	0.000	0.993
Rural	37 (48.7%)	35 (48.6%)		

### EBN competence across three assessment time points

A one-way repeated-measures ANOVA was used to examine differences in EBN competence across the three assessment time points within the Class-Clinic-OBE group. Mauchly’s test indicated that the sphericity assumption was met (*W* = 0.996, *χ²* = 0.302, *p* = 0.860). The analysis showed statistically significant differences in EBN competence across the three assessment time points [*F*(2,150) = 4.553, *p* = 0.012]. Post hoc pairwise comparisons with Bonferroni adjustment were performed to identify pairwise differences between assessment time points. The results indicated that EBN competence scores at T2 (107.50 ± 12.01) were higher than those at T0 (103.99 ± 12.30; mean difference = 3.513, *p* = 0.023) and T1 (104.14 ± 10.35; mean difference = 3.355, *p* = 0.037). However, no statistically significant difference was observed between T0 and T1 (*p* = 1.000) (Table 4).

**Table 4:** EBN competence across three assessment time points in the Class-Clinic-OBE group (*n* = 76)

Assessment time points	EBN competence mean ± SD	F	*p*
Pre-class (T0)	103.99 ± 12.30	4.553	0.012
After-The First Classroom (T1)	104.14 ± 10.35		
After-Field-Based Activities (T2)	107.50 ± 12.01		
Post hoc pairwise comparisons (Bonferroni)			
Pre-class vs. After-The First Classroom		0.1588^*^	1.000
Pre-class vs. After-Field-Based Activities		3.513^*^	0.023
After-The First Class vs. After-Field-Based Activities		3.355^*^	0.037

* represents Mean Difference

### Offline academic performance in the EBN projects

#### Performance of preliminary drafts in the Class-Clinic-OBE group

Within the first month of clinical practicum, a total of 36 preliminary drafts were submitted in the Class-Clinic-OBE group, developed through collaborative EBN project work within CoPs. Scores ranged from 88 to 97. The pass rate was 100%, with an excellence rate of 88.6% (scores ≥ 90 considered excellent). In addition, of the 36 preliminary drafts, 5.6% were submitted to academic journals.

#### Performance on proposal presentations, final presentations, and preliminary drafts in the Class-Clinic-OBE group

**Fig. 3:**
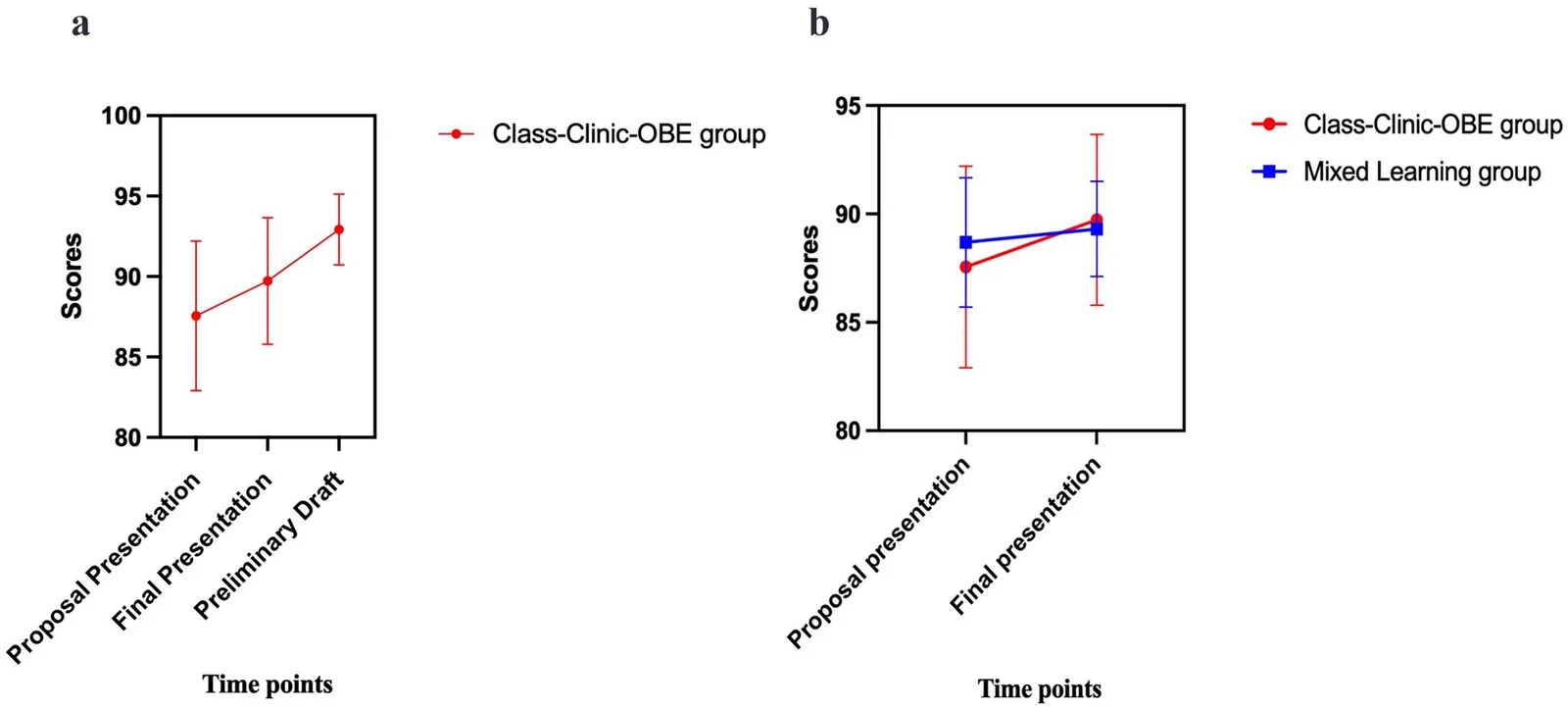
Academic performance scores across assessment time points. (**a**) Academic performance scores across three assessment time points in the Class-Clinic-OBE group. (**b**) Comparison of academic performance scores across presentation phases between groups

A one-way repeated-measures ANOVA was used to assess differences in academic performance scores across the three assessment time points. Table 5 presents the mean scores for proposal presentations, final presentations, and preliminary drafts, respectively. As shown in Fig. 3a, statistically significant differences in academic performance scores were identified across the three assessment time points [*F*(2, 156) = 43.057, *p* < 0.001].

**Table 5:** Academic performance scores online and offline in both groups

Academic Performance scores	Class-Clinic-OBE Group (*n* = 76) mean ± SD	Mixed Learning Group (*n* = 72) mean ± SD	Statistics	*p*
Online				
Grades on the Chaoxing Platform	87.76 ± 3.88	72.28 ± 2.06	*t* = -30.22	< 0.001
Offline				
Proposal Presentation	87.56 ± 4.65	88.69 ± 2.98	*F*(1,149) = 13.418	< 0.001
Final Presentation	89.73 ± 3.94	89.31 ± 2.19		
Preliminary draft	91.82 ± 2.10			

F: Repeated-measures ANOVA with statistical values of F-values

t: Independent sample t-test with statistical values of t-values

Compared to the Mixed Learning group, the Class-Clinic-OBE group progressed to clinical situations (Field-Based Activities) and produced one additional academic outcome (preliminary drafts) during this phase

#### Performance of proposal and final presentations between groups

A 2 × 2 repeated-measures ANOVA was conducted to compare differences in academic performance scores between the Class-Clinic-OBE and Mixed Learning groups across the presentation phases. The between-participant factor was group, and the within-participant factor was time (proposal presentations vs. final presentations). Results showed that academic performance scores in the Class-Clinic-OBE group were significantly higher at the final presentation than at the proposal presentation. Figure 3b visually presents the trend in score improvement. The Mixed Learning group improved by 0.62 points while the Class-Clinic-OBE group improved by 2.17 points over the same period. Although the Mixed Learning group had higher scores in proposal presentation, the Class-Clinic-OBE group demonstrated larger score differences between the two presentations. A statistically significant interaction effect between group and time was identified [*F*(1, 149) = 13.418, *p <* 0.001, *η²* = 0.083].

### Online academic performance on the Chaoxing platform

#### Engagement grades on the Chaoxing platform between groups

Tests for normality and homogeneity of variance were first conducted, and assumptions were met for both groups. Therefore, an independent-samples t-test was used to assess differences in online engagement scores between the two groups on the Chaoxing learning platform. The Class-Clinic-OBE group achieved higher total online engagement scores than the Mixed Learning group, and the between-group difference was statistically significant (*p <* 0.001).

## Discussion

Guided by the SLT and OBE theory, this quasi-experimental study evaluated the effectiveness of the Class-Clinic-OBE learning mode in the Evidence-based Nursing course for postgraduate students. Results revealed that within the Class-Clinic-OBE group, EBN competence improved across assessment time points, with higher scores observed at T2. In addition, the Class-Clinic-OBE group achieved higher scores in final presentations and online engagement compared with the Mixed Learning group. As the key learning outcome within the OBE framework, only the Class-Clinic-OBE group produced high-quality preliminary drafts.

Previous EBN educational studies have primarily focused on classroom-based instruction. In contrast, this study further incorporated Field-Based Activities and longitudinal project translation processes within authentic clinical situations. This approach extends the application of SLT and OBE frameworks in postgraduate nursing education. Under the Class-Clinic-OBE learning mode, EBN competence scores showed stage-related variation following the completion of Field-Based Activities. These findings may be interpreted in relation to Lave and Wenger’s LPP framework [12]. The framework posits that learning progresses through a gradual process of “observation, participation, and mastery”. This finding is consistent with previous conclusions on the effectiveness of SLT-guided learning, demonstrating that situated learning in authentic contexts may contribute to improvements in clinical competencies, knowledge translation, and professional identity formation in nursing education [19]. In the EBN course, a comprehensive teaching organization form emphasized supporting students’ transition from observers and learners in the classroom to practitioners in clinical situations. In the preliminary phase, students engaged in structured peripheral learning activities within “The First Classroom”. This foundational stage involved critically exploring complex clinical nursing dilemmas and formulating precise PICO (Patient, Intervention, Comparison, Outcome) questions. Through systematic literature reviews and targeted guidance from supervisors, students built a solid theoretical and methodological foundation, which is essential for the subsequent development of EBN projects. In the project execution phase, students actively applied this core EBN knowledge. They synthesized evidence tailored to predefined clinical dilemmas and translated it into actionable proposals. This process culminated in final presentations that showcased students’ ability to integrate theoretical knowledge with analytical skills. In the subsequent stage, emphasis shifted to preliminary adjustments and application during “Field-Based Activities”. Upon entering clinical internships, students implemented their EBN project outcomes in authentic clinical situations. Guided by clinical tutors and advanced practice practitioners, they refined interventions to meet real-world clinical demands and patient needs. This comprehensive participation process suggests that authentic clinical engagement, together with expanded thematic content and Field-Based Activities, may contribute to higher EBN competence scores during clinical practice. This finding may indicate the potential role of authentic clinical engagement in supporting skill development and professional identity formation, as supported by Relster et al. [31], who demonstrated that integrating evidence into clinical situations facilitates students’ knowledge translation and identity transformation. Concurrently, this process suggests the potential educational value of the “learning by doing” strategy adopted in this study, consistent with the conclusions drawn in Perez’s earlier research [32]. In summary, situational learning may play an important role in transforming EBN knowledge into practice [33]. It is imperative to offer nursing postgraduates knowledge-transfer courses through projects connected to authentic clinical situations [6].

While previous research suggests that EBN knowledge acquisition can follow a gradual progression [34], this study’s longitudinal findings suggest a potentially spiral, iterative pattern of EBN learning. Postgraduates in the Class-Clinic-OBE group initially scored lower in proposal presentations than those in the Mixed Learning group. However, they ultimately showed higher scores than the Mixed Learning group in final presentations, with larger score differences between presentations. These improvement patterns suggest that EBN learning is more than a linear accumulation of knowledge.

Rather, it may reflect an iterative learning pattern that supports the development of EBN competence through repeated cycles of “input-internalization-output”. On this spiral staircase, students first acquire foundational knowledge, such as the PICO format and literature search techniques, through classroom theoretical learning. Then, they deepen their understanding through situated learning and the formation of CoPs, subsequently identifying knowledge gaps when addressing novel clinical problems. This process may encourage further exploration and innovation, allowing EBN knowledge acquisition to develop through repeated cycles of application and reflection. In short, the spiral approach to learning goes beyond linear knowledge accumulation. It enables nursing postgraduates to develop an ongoing, evidence-based practice cycle that fosters continuous innovation in clinical decision-making and high-quality patient care.

Group work is one of the most commonly used teaching methods and is recommended as an effective strategy for enhancing EBN learning [35]. In this study, the intervention group completed 36 evidence-based projects through collaborative task division within groups, suggesting the potential educational value of learning communities. Students formed CoPs within group units, undertaking tasks such as literature searching, quality appraisal, data/evidence extraction and analysis, and final outcome presentations under targeted supervisor guidance. This collaborative approach reduces novices’ cognitive load when tackling complex research projects by fostering group interaction, which may facilitate the development of high-quality preliminary drafts. These outcomes are consistent with previous findings [36]. Small group discussions and meetings are widely recognized as efficient and readily implementable communication strategies. Face-to-face communication enhances interaction and learning among participants, offering academic-practice EBN collaborators dedicated time and space to focus on EBN learning [37]. This process may also align with the OBE-oriented goal of enhancing academic productivity. Furthermore, the dynamic assessment mechanism in longitudinally tracking preliminary draft revisions may create a continuous feedback loop. Students refine research designs through multiple rounds of mentor feedback, aligning their empirical work with the skill-dimension cultivation objectives in the EBP-COQ questionnaire [38].

This study demonstrates that clearly defined learning objectives grounded in the OBE framework may facilitate the enhancement of students’ EBN competence. This finding aligns with existing evidence on the effectiveness of OBE learning. A systematic review has confirmed that OBE-based teaching has been associated with improvements in nursing students’ theoretical knowledge, clinical skills, self-directed learning, and critical thinking [39]. These carefully designed objectives emphasize the crucial process of translating evidence-based theory into practice through EBN projects. These projects allow students to engage deeply with their subjects and apply acquired knowledge in authentic clinical settings. Ultimately, the main output of EBN projects is an evidence-based preliminary draft that demonstrates students have internalized EBN knowledge and can translate it into actionable solutions. In summary, applying OBE principles to define teaching objectives in EBN courses may improve students’ implementation skills and support outcomes more closely aligned with clinical needs.

Besides, the Class-Clinic-OBE group demonstrated superior performance on the Chaoxing online learning platform. These differences may be related to the challenge-based task-point delivery mode, which may encourage student engagement, combined with instant feedback and progress tracking, suggesting that task-based gamification may support participation and competence development in EBN education [40].

### Limitations

Although the Class-Clinic-OBE learning mode demonstrated some promising effects, this study has several limitations. First, the study employed a non-concurrent two-cohort quasi-experimental design without randomization, which limits causal inference. While baseline demographic characteristics were comparable (Table 3), temporal confounding and external factors cannot be completely excluded. In addition, compared with the Mixed Learning group, the Class-Clinic-OBE group received expanded thematic content and Field-Based Activities within authentic clinical situations. Therefore, the observed differences may have been influenced by multiple interconnected educational components and environmental changes. Second, the single-center sample limits the generalizability of the findings to other educational institutions with different clinical cultures or resource levels, potentially obscuring how CoP dynamics vary across settings. Third, EBN competence was measured through a participant self-reporting instrument (EBP-COQ). Self-report measures may not fully capture nuanced behavioral manifestations of EBN competence in actual clinical practice. To complement this, we also included objective performance measures (online engagement data, proposal and final presentation scores, and preliminary draft quality), which provide a more comprehensive picture of students’ abilities. Fourth, academic performance was assessed using self-made grading scales that did not undergo expert validity review or inter-rater reliability testing. Although scoring consistency was strengthened through calibration discussions, consensus meetings, and trial scoring procedures, potential subjectivity in assessment could not be fully excluded. Fifth, the lack of long-term tracking beyond the program period prevents evaluation of whether EBN competence can be sustained in authentic clinical situations over time.

## Conclusion

EBN competence is essential for postgraduate nursing students. They should be thoroughly prepared for it through both classroom learning and clinical practicum so that they are fully competent before assuming hospital positions. The findings suggest that integrating situated learning with outcome-based education may facilitate cross-context learning between classroom and clinical practice. This approach provides a potentially feasible framework for strengthening EBN education in postgraduate nursing curriculum.

## Supplementary Information

Below is the link to the electronic supplementary material.

Supplementary Material 1

Supplementary Material 2

Supplementary Material 3

## Data Availability

The datasets generated and analyzed during the current study are not publicly available due to privacy and confidentiality reasons, but are available from the corresponding author on reasonable request.

## Abbreviations

EBN: Evidence-based Nursing
EBP-COQ: Evidence-Based Practice Competence Questionnaire
OBE: Outcome-Based Education
SLT: Situated Learning Theory
CoPs: Communities of Practice
TBL: Team-Based Learning
PBL: Problem-Based Learning
CBL: Case-Based Learning
ChBL: Challenge-Based Learning
SPOC: A Small Private Online Course
PICO: Patient, Intervention, Comparison, Outcome
RevMan: Review Manager
SCI: Science Citation Index
