# Supplementary material for: Building evidence-based nursing competence through Class-Clinic-OBE in postgraduate education: a quasi-experimental study
Source: BMC Nurs. 2026 Jul 28;25:1086. doi: 10.1186/s12912-026-04981-6 (PMC13746706; doi:10.1186/s12912-026-04981-6)
Supplement: Supplementary file 1 [file 12912_2026_4981_MOESM2_ESM.pdf]

Table 1: Proposal Presentation Rubric for Meta-Analysis of Quantitative Research.

Assessment Domain	Assessment Criterion	Scoring Criteria	Points
**I. Topic Selection and Clinical Relevance (40 points)**	Clinical Problem and Practice Background	The topic originates from a real clinical problem and has a clear practice background and nursing significance.	10
	Innovativeness and Research Value	The topic demonstrates some innovativeness and addresses an evidence gap in current nursing practice.	10
	Evidence- Based Significance	There is controversy in existing evidence or a clear need for practice improvement, justifying an EBN project.	20
**II. EBN Question Formulation (20 points)**	PICO Construction	The PICO elements (Population, Intervention, Comparison, Outcome) are complete, reasonable, and the research question is clearly stated.	10
	Population and Outcome Definition	The target population, intervention(s), and outcome measures are clearly defined and measurable.	10
**III. Literature Search and Study Design (20 points)**	Search Strategy	Search terms are standardized, databases are appropriately selected, and the search process is complete.	10
	Search Strategy Presentation	Full search strategies for at least one Chinese and one English database are properly displayed.	10
**IV. Clinical Translation Potential and Collaboration (15 points)**	Clinical Translation Potential	The research question has clear clinical applicability; the findings may inform nursing practice improvement.	10
	Team Collaboration	Division of labour is reasonable and all members participate actively.	5
**V. Presentation and Expression (5 points)**	Logic and Clarity	The presentation is well- structured, clear, and fluent.	3
	PPT and Time Management	Slides are well- designed and the presentation fits the allocated time.	2
**Total Score**			100

Table 2: Proposal Presentation Rubric for Meta-Synthesis of Qualitative Research.

Assessment Domain	Assessment Criterion	Scoring Criteria	Points
**I. Topic Selection and Clinical Relevance (40 points)**	Clinical Problem and Practice Background	The research topic originates from a real nursing practice problem and has clinical significance.	10
	Innovativeness and Research Value	The topic demonstrates some innovativeness and practical value.	10
	Suitability for Meta-Synthesis	The phenomenon of interest has sufficient qualitative evidence to justify a meta-synthesis.	20
**II. EBN Question Formulation (20 points)**	PICo Construction	The PICo elements (Population, phenomenon of Interest, Context) are reasonably constructed and the question is clear.	10
	Phenomenon and Context Definition	The phenomenon of interest and the research context are clearly defined.	10
**III. Literature Search and Qualitative Synthesis (20 points)**	Search Strategy	Search terms are standardised, databases are appropriately selected, and the search process is complete.	10
	Search Strategy Presentation	Full search strategy for at least one database is properly displayed.	10
**IV. Practice Implications and Collaboration (15 points)**	Practice Implications	The findings may inform nursing practice, holistic care, or patient support in the future.	10
	Team Collaboration	Division of labour is reasonable and all members participate actively.	5
**V. Presentation and Expression (5 points)**	Logic and Clarity	The presentation is well-structured, clear, and fluent.	3
	PPT and Time Management	Slides are well-designed and the presentation fits the allocated time.	2
**Total Score**			100

Table 3: Proposal Presentation Rubric for Summary of Best Available Evidence.

Assessment Domain	Assessment Criterion	Scoring Criteria	Points
**I. Topic Selection and Clinical Relevance (40 points)**	Clinical Problem and Practice Background	The topic originates from a real nursing practice problem and has potential for evidence-based application.	10
	Innovativeness and Research Value	The topic shows some practical innovativeness or value for quality improvement.	10
	Evidence-Based Significance	There is a need for evidence-based practice, and the topic is suitable for a best evidence summary.	20
**II. EBN Question Formulation (20 points)**	PIPOST Construction	The PIPOST elements are reasonably constructed and the question is clearly defined.	10
	Clinical Context and Target Population	The practice setting, target population, and outcome indicators are clearly stated.	10
**III. Literature Search and Evidence Synthesis (20 points)**	Search Strategy	Search terms are standardised, databases are appropriately selected, and the search process follows the “6S” pyramid (top-down).	10
	Search Strategy Presentation	Full search strategies for at least one Chinese and one English database are properly displayed.	10
**IV. Evidence Translation Potential and Collaboration (15 points)**	Evidence Translation Potential	The summarised evidence has potential for future evidence-based practice dissemination and nursing quality improvement.	10
	Team Collaboration	Division of labour is reasonable and all members participate actively.	5
**V. Presentation and Expression (5 points)**	Logic and Clarity	The presentation is well-structured, clear, and fluent.	3
	PPT and Time Management	Slides are well-designed and the presentation fits the allocated time.	2
**Total Score**			100

Table 4: Final Presentation Rubric for Meta-Analysis of Quantitative Research.

Assessment Domain	Assessment Criterion	Scoring Criteria	Points
**I. Literature Quality Appraisal (15 points)**	Literature Screening Process	Independent screening or spot-checking demonstrated a consistency of ≥0.8; a PRISMA flow diagram was provided.	7
	Risk of Bias Assessment	An appropriate risk-of-bias assessment tool was used; independent double assessment was confirmed; results were presented clearly.	8
**II. Data Extraction (10 points)**	Data Extraction from Included Studies	Independent double data extraction or spot-checking demonstrated a consistency of ≥0.8; standardized extraction templates were used.	5
	List of Excluded Studies	A list of excluded studies with rationales was provided.	5
**III. Statistical Analysis (20 points)**	Effect Size and Statistical Methods	Select an appropriate effect size and statistical model (fixed/random effects).	5
	Heterogeneity Assessment	Sources of heterogeneity were investigated (e.g., subgroup/sensitivity analysis) and addressed appropriately.	5
	Impact of Risk of Bias on Pooled Results	The potential impact of risk of bias in included studies on the overall effect was considered.	5
	Heterogeneity and Publication Bias Assessment	Sources of heterogeneity were explored, and their impact on findings was discussed. Publication bias was assessed via graphical/statistical tests, with its likelihood and impact on results discussed.	5
**IV. Results and Discussion (45 points)**	Characteristics of Included Studies	A detailed description of included study characteristics (participants, interventions, comparators, outcomes, study design, follow-up duration, etc.) was provided.	6
	Research Protocol and Registration	Whether the protocol was pre-specified, registered, or published was clearly stated.	6

Table 4 (continued)

Assessment Domain	Assessment Criterion	Scoring Criteria	Points
**IV. Results and Discussion (45 points)**	Conflict of interest	State any potential conflicts of interest.	6
	Methodological Consistency	The systematic review was conducted in strict accordance with PRISMA or Cochrane guidelines.	12
	Conclusion Robustness	Conclusions were appropriately cautious, with recommendations aligned to the strength of evidence.	15
**V. Presentation and Expression (10 points)**	Logic and Clarity	The presentation is logically structured with key points clearly highlighted.	5
	Language and Time Management	Communication is clear and fluent, and the presentation adheres to the allotted time.	3
	Slide Design	Slides are concise, well-organized, and visually clear, with legible charts and figures.	2
**Total Score**			100

Table 5: Final Presentation Rubric for Meta-Synthesis of Qualitative Research.

Assessment Domain	Assessment Criterion	Scoring Criteria	Points
**I. Literature Quality Appraisal (15 points)**	Quality Appraisal Tool	Specify the quality appraisal tools used (e.g., CASP, JBI, etc.).	5
	Quality Appraisal Process	Quality appraisal was conducted independently by two reviewers, with a clear process for reaching consensus.	5
	Quality Appraisal Results	Present quality appraisal results and document any studies excluded due to poor methodological quality.	5
**II. Data Extraction (10 points)**	Data Extraction	Describe the data extracted from included studies (e.g., themes, citations, contextual details) and the extraction process used.	10
**III. Analysis and Integration (20 points)**	Findings Synthesis	Present novel interpretations, conceptual models, theories, or thematic frameworks that transcend individual study-level findings.	15
	Methodological Consistency	The synthesis strictly followed the chosen methodology, with a transparent and auditable process.	5
**IV. Results and Discussion (45 points)**	Reflexivity	Reflect on the researchers′ potential biases and their influence on the synthesis findings.	10
	Interpretation of Findings	Interpret the synthesis findings in the context of existing literature or relevant theories.	15
	Practice Implications	Provide specific recommendations for nursing practice and patient care	20
**V. Presentation and Expression (10 points)**	Logic and Clarity	The presentation is logically structured with key points clearly highlighted.	5
	Language and Time Management	Communication is clear and fluent, and the presentation adheres to the allotted time.	3
	Slide Design	Slides are concise, well-organized, and visually clear, with legible charts and figures.	2
**Total Score**			100

Table 6: Final Presentation Rubric for Summary of Best Available Evidence

Assessment Domain	Assessment Criterion	Scoring Criteria	Points
**I. Literature Quality Appraisal (15 points)**	Strengths and Limitations of Included Evidence	Assess and report the strengths and limitations of included evidence (quality, quantity, and consistency).	4
	Evidence Grading	Clearly state the use of GRADE or JBI evidence grading systems and report grading results.	5
	Process Transparency and Reproducibility	The evidence summary process is fully reproducible and transparent.	4
	External Review	The evidence summary underwent external peer review prior to finalization.	2
**II. Data Extraction (10 points)**	Inclusion/Exclusion Criteria	Clear inclusion and exclusion criteria were defined and reported.	5
	Alignment Between Recommendations and Evidence	There is a clear link between each recommendation and its supporting evidence.	5
**III. Statistical Analysis (20 points)**	Recommendation Development Process	Describe the methods used to develop recommendations (e.g., expert consensus, GRADE framework).	12
	Benefits, Harms, and Risks Consideration	Health benefits, adverse effects, and risks were considered when formulating recommendations.	8
**IV. Results and Discussion (45 points)**	Multidisciplinary team	The development team included relevant stakeholders (e.g., clinicians, methodologists, and patient representatives).	4
	Target Population Perspectives	Patient preferences and values were considered.	3
	Target Users	Target users (e.g., nurses, physicians, managers) were clearly defined.	3
	Clarity of Recommendations	Each recommendation is clear and specific.	4
	Alternative Management Options	Different management options were presented for each clinical question.	3

Table 6 (continued)

Assessment Domain	Assessment Criterion	Scoring Criteria	Points
**IV. Results and Discussion (45 points)**	Identifiability of Main Recommendations	The main recommendations should be clear and easy to read (e.g., in bold or highlighted).	3
	Facilitators and Barriers to Implementation	Potential facilitators and barriers to implementation were described.	4
	Implementation Tools and Resources	Provide practical recommendations or supporting tools (such as flowcharts and checklists).	4
	Resource Investment	Resources required to implement the recommendations (e.g., personnel, equipment, funding) were considered.	4
	Monitoring and Review Standards	Clear standards for monitoring and reviewing the implementation of recommendations were defined.	3
**IV. Results and Discussion (45 points)**	The Impact of Sponsoring Organizations	The sponsor's influence did not bias or affect the content of the evidence summary.	5
	conflict of interest	Conflicts of interest among team members were recorded and appropriately managed.	5
	Update Plan	A clear plan or schedule for updating the evidence summary was provided.	2
**V. Presentation and Expression (10 points)**	Logic and Clarity	The presentation is logically structured with key points clearly highlighted.	5
	Language and Time Management	Communication is clear and fluent, and the presentation adheres to the allotted time.	3
	Slide Design	Slides are concise, well-organized, and visually clear, with legible charts and figures.	2
**Total score**			100

Notes:

This rubric was used to assess proposal and final presentations in both the Mixed Learning and Class-Clinic-OBE groups.

Each project group was evaluated by one designated faculty supervisor (sole rater).

Scores should be assigned according to the descriptors provided for each criterion.

All criteria and weighting were communicated to students before assessment.

The total score is calculated as the weighted sum of all assessment domains.
