# Supplementary material for: Building evidence-based nursing competence through Class-Clinic-OBE in postgraduate education: a quasi-experimental study
Source: BMC Nurs. 2026 Jul 28;25:1086. doi: 10.1186/s12912-026-04981-6 (PMC13746706; doi:10.1186/s12912-026-04981-6)
Supplement: Supplementary file 2 [file 12912_2026_4981_MOESM3_ESM.pdf]

Table 1: Preliminary Draft Rubric for Meta-Analysis of Quantitative Research.

Assessment Domain	Assessment Criterion	Scoring Criteria	Points
**I. Research Background & EBN Question Formulation (10 points)**	Research Background & Evidence Gap	The research background and current status of the field are systematically reviewed. Evidence gaps are accurately identified, demonstrating problem- orientation and clinical relevance.	5
	EBN Question Formulation	The EBN question is appropriately constructed using a PICO/PICo/PIO/PIPOST framework, is logically clear, and has clinical practice value.	5
**II. Literature Search & Research Methodology (20 points)**	Literature Sources & Search Strategy	Databases are appropriately selected. The search strategy is clear and complete, including search string examples, date ranges, and justifications for any restrictions.	5
	Literature Screening & Quality Appraisal	Inclusion/exclusion criteria are clearly defined. The screening process is properly presented. Appropriate appraisal tools are used, and results are explained.	10
	Rigour of Research Methods	The study design and methods are fully described, with a clear research process. Evidence synthesis methods, statistical approaches, or implementation processes are appropriately explained, demonstrating rigour, transparency, and reproducibility.	5
**III. Evidence Extraction & Result Analysis (15 points)**	Evidence Extraction	Standardised tables are used to present the characteristics of included studies. Key information is complete without notable omissions.	5
	Data Analysis & Evidence Synthesis	Research findings are clearly presented, and statistical or evidence synthesis results are correctly interpreted. Similarities and differences across studies are compared, potential reasons are analysed, and a certain level of evidence- based analytical ability is demonstrated.	10
**IV. Discussion, Evidence Translation & Critical Analysis (20 points)**	Interpretation & Theoretical Linkage	Results are analysed in depth by linking to theoretical mechanisms or related studies, reflecting evidence- based thinking.	5

Table 1 (continued)

Assessment Domain	Assessment Criterion	Scoring Criteria	Points
**IV. Discussion, Evidence Translation & Critical Analysis (20 points)**	Critical Analysis Ability	Systematic reflection on methodological quality, evidence strength, and study limitations is provided, going beyond simple description.	5
	Clinical Application & Evidence Translation	The implications of the findings for clinical nursing practice and the value for evidence translation are clearly articulated.	5
	Innovativeness & Academic Value	The study demonstrates novelty, practical significance, or potential academic contribution.	5
**V. Conclusions & Future Directions (10 points)**	Research Conclusions	Conclusions accurately address the research questions, are logically rigorous, and avoid over- interpretation.	5
	Suggestions & Future Directions	Reasonable and feasible clinical recommendations or future research directions are proposed.	5
**VI. References & Academic Standards (15 points)**	Quality & Quantity of References	References are from standard sources, preferentially citing high- quality evidence- based literature, with sufficient quantity.	5
	Accuracy of Citations	Citations appropriately support the research questions and follow academic formatting standards.	5
	Academic Standards & Formatting	Tables and figures are presented properly with complete titles and numbering; formatting adheres to academic paper requirements.	5
**VII. Paper Structure & Language Expression (10 points)**	Structure & Logic	The content is well- organised, the argumentation is logical, and transitions between sections are natural.	5
	Language Expression	Academic language is standard, accurate, and logically rigorous, without obvious grammatical errors or ambiguity.	5
**Total Score**			100

Table 2: Preliminary Draft Rubric for Meta-Synthesis of Qualitative Research.

Assessment Domain	Assessment Criterion	Scoring Criteria	Points
**I. Research Background & EBN Question Formulation (10 points)**	Research Background & Evidence Gap	The research background and current status of the field are systematically reviewed. Evidence gaps are accurately identified, demonstrating problem- orientation and clinical relevance.	5
	EBN Question Formulation	The EBN question is appropriately constructed using a PICO/PICo/PIO/PIPOST framework, is logically clear, and has clinical practice value.	5
**II. Literature Search & Research Methodology (20 points)**	Literature Sources & Search Strategy	Databases are appropriately selected. The search strategy is clear and complete, including search string examples, date ranges, and justifications for any restrictions.	5
	Literature Screening & Quality Appraisal	Inclusion/exclusion criteria are clearly defined. The screening process is properly presented. Appropriate appraisal tools (e.g., CASP, JBI) are used, and results are explained.	10
	Rigour of Research Methods	The study design and methods are fully described, with a clear research process. Evidence synthesis methods (e.g., thematic synthesis, meta- ethnography) or implementation processes are appropriately explained, demonstrating rigour, transparency, and reproducibility.	5
**III. Evidence Extraction & Result Analysis (15 points)**	Evidence Extraction	Standardised tables are used to present the characteristics of included studies. Key information is complete without notable omissions.	5
	Data Analysis & Evidence Synthesis	Research findings are clearly presented, and evidence synthesis results are correctly interpreted. Similarities and differences across studies are compared, potential reasons are analysed, and a certain level of evidence- based analytical ability is demonstrated.	10
**IV. Discussion, Evidence Translation & Critical Analysis (20 points)**	Interpretation & Theoretical Linkage	Results are analysed in depth by linking to theoretical mechanisms or related studies, reflecting evidence- based thinking.	5
	Critical Analysis Ability	Systematic reflection on methodological quality, evidence strength, and study limitations is provided, going beyond simple description.	5

Table 2 (continued)

Assessment Domain	Assessment Criterion	Scoring Criteria	Points
**IV. Discussion, Evidence Translation & Critical Analysis (20 points)**	Clinical Application & Evidence Translation	The implications of the findings for clinical nursing practice and the value for evidence translation are clearly articulated.	5
	Innovativeness & Academic Value	The study demonstrates novelty, practical significance, or potential academic contribution.	5
**V. Conclusions & Future Directions (10 points)**	Research Conclusions	Conclusions accurately address the research questions, are logically rigorous, and avoid over- interpretation.	5
	Suggestions & Future Directions	Reasonable and feasible clinical recommendations or future research directions are proposed.	5
**VI. References & Academic Standards (15 points)**	Quality & Quantity of References	References are from standard sources, preferentially citing high- quality evidence- based literature, with sufficient quantity.	5
	Accuracy of Citations	Citations appropriately support the research questions and follow academic formatting standards.	5
	Academic Standards & Formatting	Tables and figures are presented properly with complete titles and numbering; formatting adheres to academic paper requirements.	5
**VII. Paper Structure & Language Expression (10 points)**	Structure & Logic	The content is well- organised, the argumentation is logical, and transitions between sections are natural.	5
	Language Expression	Academic language is standard, accurate, and logically rigorous, without obvious grammatical errors or ambiguity.	5
**Total Score**			100

Table 3: Preliminary Draft Rubric for Summary of Best Available Evidence.

Assessment Domain	Assessment Criterion	Scoring Criteria	Points
**I. Research Background & EBN Question Formulation (10 points)**	Research Background & Evidence Gap	The research background and current status of the field are systematically reviewed. Evidence gaps are accurately identified, demonstrating problem- orientation and clinical relevance.	5
	EBN Question Formulation	The EBN question is appropriately constructed using a PICO/PICo/PIO/PIPOST framework, is logically clear, and has clinical practice value.	5
**II. Literature Search & Research Methodology (20 points)**	Literature Sources & Search Strategy	Databases are appropriately selected. The search strategy is clear and complete, including search string examples, date ranges, and justifications for any restrictions.	5
	Literature Screening & Quality Appraisal	Inclusion/exclusion criteria are clearly defined. The screening process is properly presented. Appropriate appraisal tools are used, and results are explained.	10
	Rigour of Research Methods	The study design and methods are fully described, with a clear research process. Evidence synthesis methods (e.g., GRADE, JBI) or implementation processes are appropriately explained, demonstrating rigour, transparency, and reproducibility.	5
**III. Evidence Extraction & Result Analysis (15 points)**	Evidence Extraction	Standardised tables are used to present the characteristics of included studies. Key information is complete without notable omissions.	5
	Data Analysis & Evidence Synthesis	Research findings are clearly presented, and evidence synthesis results are correctly interpreted. Similarities and differences across studies are compared, potential reasons are analysed, and a certain level of evidence- based analytical ability is demonstrated.	10
**IV. Discussion, Evidence Translation & Critical Analysis (20 points)**	Interpretation & Theoretical Linkage	Results are analysed in depth by linking to theoretical mechanisms or related studies, reflecting evidence- based thinking.	5
	Critical Analysis Ability	Systematic reflection on methodological quality, evidence strength, and study limitations is provided, going beyond simple description.	5

Table 3 (continued)

Assessment Domain	Assessment Criterion	Scoring Criteria	Points
**IV. Discussion, Evidence Translation & Critical Analysis (20 points)**	Clinical Application & Evidence Translation	The implications of the findings for clinical nursing practice and the value for evidence translation are clearly articulated.	5
	Innovativeness & Academic Value	The study demonstrates novelty, practical significance, or potential academic contribution.	5
**V. Conclusions & Future Directions (10 points)**	Research Conclusions	Conclusions accurately address the research questions, are logically rigorous, and avoid over- interpretation.	5
	Suggestions & Future Directions	Reasonable and feasible clinical recommendations or future research directions are proposed.	5
**VI. References & Academic Standards (15 points)**	Quality & Quantity of References	References are from standard sources, preferentially citing high- quality evidence- based literature, with sufficient quantity.	5
	Accuracy of Citations	Citations appropriately support the research questions and follow academic formatting standards.	5
	Academic Standards & Formatting	Tables and figures are presented properly with complete titles and numbering; formatting adheres to academic paper requirements.	5
**VII. Paper Structure & Language Expression (10 points)**	Structure & Logic	The content is well- organised, the argumentation is logical, and transitions between sections are natural.	5
	Language Expression	Academic language is standard, accurate, and logically rigorous, without obvious grammatical errors or ambiguity.	5
**Total Score**			100

Notes:

This rubric was used to assess preliminary drafts only in the Class-Clinic-OBE groups.

Each project group was evaluated by one designated faculty supervisor (sole rater).

Scores should be assigned according to the descriptors provided for each criterion.

All criteria and weighting were communicated to students before assessment.

The total score is calculated as the weighted sum of all assessment domains.
