# Supplementary material for: Building evidence-based nursing competence through Class-Clinic-OBE in postgraduate education: a quasi-experimental study
Source: BMC Nurs. 2026 Jul 28;25:1086. doi: 10.1186/s12912-026-04981-6 (PMC13746706; doi:10.1186/s12912-026-04981-6)
Supplement: Supplementary file 3 [file 12912_2026_4981_MOESM1_ESM.docx]

**Appendix 1** Evidence-Based Practice Competency Questionnaire (EBP-COQ) – English version.

| Item | English Version (Original) |
| --- | --- |
| 1 | The EBP helps to make decisions in clinical practice. |
| 2 | I’m confident that I will be able to evaluate critically the quality of a scientific article. |
| 3 | The practice of EBP will help to have a better definition of the nurse roll. |
| 4 | The nursing contract should include time to read scientific papers and make critical appraisal of them. |
| 5 | The widespread EBP implementation will allow to increase nursing autonomy from others professions. |
| 6 | When I work as a nurse I will pleased if the EBP will be in practice. |
| 7 | The application of EBP improves patient's health care outcomes. |
| 8 | In the future I wish to contribute to apply the EBP. |
| 9 | I do not like reading scientific articles. |
| 10 | The patient care will experiment minor changes with the EBP application. |
| 11 | It pleased me that the EBP is only a theoretical movement that does not takes in practice. |
| 12 | If I will have the opportunity I would assist to an EBP course. |
| 13 | I would like to have better access to published nursing scientific evidences. |
| 14 | I feel able to make a clinical question to start the searching of the best scientific evidence. |
| 15 | I do not feel able to search for scientific evidences in the principles heath sciences data bases. |
| 16 | I do not feel able to search for the scientific information about the subject in the most important bibliographic indexes. |

**Appendix 1** (continued)

| 17 | I feel able to evaluate critically the quality of a scientific article. |
| --- | --- |
| 18 | I do not feel able to analyze if the obtained results of a scientific study are valid. |
| 19 | I feel able to analyze the practical utility of a scientific study. |
| 20 | I know how to make clinical questions organize in the PICO format. |
| 21 | I know the principal sources that offer the information revised and catalogued behind the evidence point of view. |
| 22 | I do not know the most important characteristics of the principal investigation designs. |
| 23 | I know the different evidence level of the designs of the investigation studies. |
| 24 | I do not know the different recommendation grades about the adoption of a determined procedure or health intervention. |
| 25 | I know the principal measures of association and potential impact that allow to evaluate the magnitude of the analyzed effect in investigation studies. |

The 25-item questionnaire includes three distinct dimensions: attitude (1-13), skills (14-19), and knowledge(20-25). Each item uses a 5-point Likert scale ranging from 1 (strongly disagree) to 5 (strongly agree), yielding a total possible score between 25 and 125. Higher scores indicate better EBN competence.
